# Supplementary material for: Novel Driver Strength Index highlights important cancer genes in TCGA PanCanAtlas patients
Source: PeerJ. 2022 Aug 11;10:e13860. doi: 10.7717/peerj.13860 (PMC9375969; doi:10.7717/peerj.13860)
Supplement: Supplemental Information 2 [file peerj-10-13860-s002.zip › COHORTS/genes plots/cohorts/2021_8_16_14_9_distribution_cohorts_males_LUAD.pdf]

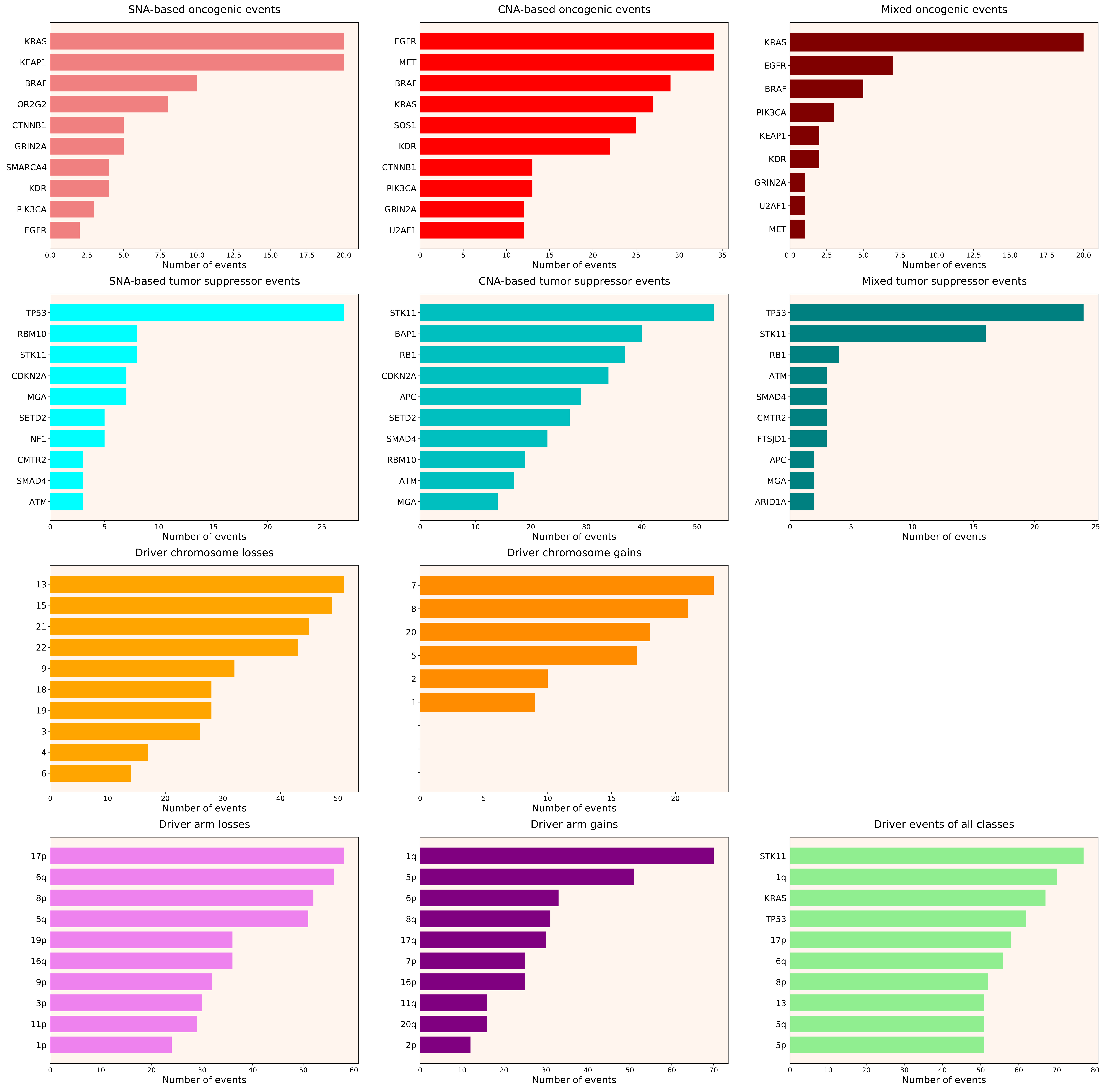
SNA-based oncogenic events
KRAS
KEAP1
BRAF
OR2G2
CTNNB1
GRIN2A
SMARCA4
KDR
PIK3CA
EGFR
0.0 2.5 5.0 7.5 10.0 12.5 15.0 17.5 20.0
Number of events
CNA-based oncogenic events
EGFR
MET
BRAF
KRAS
SOS1
KDR
CTNNB1
PIK3CA
GRIN2A
U2AF1
0 5 10 15 20 25 30 35
Number of events
Mixed oncogenic events
KRAS
EGFR
BRAF
PIK3CA
KEAP1
KDR
GRIN2A
U2AF1
MET
0.0 2.5 5.0 7.5 10.0 12.5 15.0 17.5 20.0
Number of events
SNA-based tumor suppressor events
TP53
RBM10
STK11
CDKN2A
MGA
SETD2
NF1
CMTR2
SMAD4
ATM
0 5 10 15 20 25
Number of events
CNA-based tumor suppressor events
STK11
BAP1
RB1
CDKN2A
APC
SETD2
SMAD4
RBM10
ATM
MGA
0 10 20 30 40 50
Number of events
Mixed tumor suppressor events
TP53
STK11
RB1
ATM
SMAD4
CMTR2
FTSJD1
APC
MGA
ARID1A
0 5 10 15 20 25
Number of events
Driver chromosome losses
13
15
21
22
9
18
19
3
4
6
0 10 20 30 40 50
Number of events
Driver chromosome gains
7
8
20
5
2
1
0 5 10 15 20
Number of events
Driver arm losses
17p
6q
8p
5q
19p
16q
9p
3p
11p
1p
0 10 20 30 40 50 60
Number of events
Driver arm gains
1q
5p
6p
8q
17q
7p
16p
11q
20q
2p
0 10 20 30 40 50 60 70
Number of events
Driver events of all classes
STK11
1q
KRAS
TP53
17p
6q
8p
13
5q
5p
0 10 20 30 40 50 60 70 80
Number of events
